# Mathematical Modelling for the Role of CD4^+^T Cells in Tumor-Immune Interactions

**DOI:** 10.1155/2020/7187602

**Published:** 2020-02-19

**Authors:** Ahmed M. Makhlouf, Lamiaa El-Shennawy, Hesham A. Elkaranshawy

**Affiliations:** ^1^Department of Engineering Mathematics and Physics, Faculty of Engineering, Alexandria University, Alexandria, Egypt; ^2^Institute of Graduate Studies and Research, Alexandria University, Alexandria, Egypt

## Abstract

Mathematical modelling has been used to study tumor-immune cell interaction. Some models were proposed to examine the effect of circulating lymphocytes, natural killer cells, and CD8^+^T cells, but they neglected the role of CD4^+^T cells. Other models were constructed to study the role of CD4^+^T cells but did not consider the role of other immune cells. In this study, we propose a mathematical model, in the form of a system of nonlinear ordinary differential equations, that predicts the interaction between tumor cells and natural killer cells, CD4^+^T cells, CD8^+^T cells, and circulating lymphocytes with or without immunotherapy and/or chemotherapy. This system is stiff, and the Runge–Kutta method failed to solve it. Consequently, the “Adams predictor-corrector” method is used. The results reveal that the patient's immune system can overcome small tumors; however, if the tumor is large, adoptive therapy with CD4^+^T cells can be an alternative to both CD8^+^T cell therapy and cytokines in some cases. Moreover, CD4^+^T cell therapy could replace chemotherapy depending upon tumor size. Even if a combination of chemotherapy and immunotherapy is necessary, using CD4^+^T cell therapy can better reduce the dose of the associated chemotherapy compared to using combined CD8^+^T cells and cytokine therapy. Stability analysis is performed for the studied patients. It has been found that all equilibrium points are unstable, and a condition for preventing tumor recurrence after treatment has been deduced. Finally, a bifurcation analysis is performed to study the effect of varying system parameters on the stability, and bifurcation points are specified. New equilibrium points are created or demolished at some bifurcation points, and stability is changed at some others. Hence, for systems turning to be stable, tumors can be eradicated without the possibility of recurrence. The proposed mathematical model provides a valuable tool for designing patients' treatment intervention strategies.

## 1. Introduction

Cancer is one of the leading causes of death worldwide. According to the World Health Organization (WHO), there were 8.8 million deaths in 2015 due to cancer [1]. The global cancer burden is expected to rise to nearly 21.4 million cases and 13.5 million deaths by 2030 [2]. Cancer treatment includes surgery, radiation [3], hormonal therapy [4], virotherapy [5], chemotherapy, and recently immunotherapy [6]. Chemotherapy is a well-known method for treatment of cancer. It is based on the administration of drugs that can kill tumor cells. These drugs do not only kill tumor cells but can also kill normal cells. Thus, many patients suffer from side effects of treatment as well as resistance to therapy and recurrence [7].

New approaches to treatment have been investigated, and immunotherapy has been recently approved for the treatment of many types of cancers [8, 9]. Immunotherapy is based on enhancing the effectiveness of the immune system to identify and kill tumor cells using two strategies: (1) passive immunotherapy, where effector components of the immune system are used to directly attack tumor cells. This strategy includes antibody-targeted therapy and genetically engineered T cells (e.g., chimeric antigen receptor [CAR]-T). (2) Active immunotherapy, where the activity of the immune system is enhanced. This includes cancer vaccines, cytokines, and adoptive cell therapy [10]. Cancer vaccines enhance cytotoxic T lymphocytes response to specific antigens produced by the tumor cells. Cytokines are proteins important for cell signalling and are produced by many cells, such as macrophages, B lymphocytes, and T lymphocytes. However, not all cytokines are approved for the treatment of cancer. The Food and Drug Administration (FDA) in USA approved only two cytokines, interleukin-2 (IL-2) and interferon alpha (IFN-*α*), since they demonstrated moderate clinical benefit [11]. IL-2 is most commonly used to trigger the immune system by activating T-cells and natural killer cells that identify and kill tumor cells. INF-*α* has a similar response rate to IL-2, but it does not achieve long-term patient survival compared to IL-2 [2, 10, 12, 13]. Adoptive cell therapy (ACT) involves ex vivo stimulation and expansion of tumor infiltrated T cells, then infusing the cells back into cancer patients [10]. The majority of ACT clinical trials and animal studies comprise CD4^+^T helper 1 cells and CD8^+^ cytotoxic T cells [14].

Mathematical modelling provides a powerful tool to describe and analyse many engineering and physical problems. It has also been used to describe some biological processes such as heart beats [15], diffusion of drugs [15], hepatitis C virus [16], management of HIV infection [17], treatment of diabetes [18], clearing the antibiotic resistant infection [19], aortic aneurysm formation [20], and tumor growth and cancer treatment. Studies in the field of cancer biology focus on developing suitable mathematical models involving quantitative approaches to understand various aspects of tumor growth and the response of cancer cells to clinical interventions. Jones et al. proposed a model describing tumor growth due to its internal pressure [15]. The model was in the form of a system of partial differential equations and included tumor size and internal pressure and nutrients' concentration without any treatment. Similar models, in the form of systems of partial differential equations, were also proposed by Tao et al. [21], Wei and Cui [22], Wise et al. [23], Frieboes et al. [24], Lee et al. [25], Zhang et al. [26], Knopoff et al. [27], de Pillis et al. [28], Villasana and Radunskaya [29], and Xu and Bai [30]. The last two models involved time delayed equations as well. A statistical model for cancer mortality has been studied by Ghosh and Samanta [31].

An important division of the mathematical models about cancer intends to investigate the effect of varying the concentrations of immunotherapy and chemotherapy and to explore the influence of various parameters. Valuable and comprehensive reviews can be found in [32–35]. Most of these models are in the form of a system of coupled nonlinear ordinary differential equations (see, for example, references [12, 36–39]). Along this line, de Pillis and colleagues developed a useful model for the interaction between the tumor and immune system [13, 40–43]. They obtained a numerical solution and performed both stability analysis and sensitivity analysis. They took into account the following assumptions: (a) a tumor grows logistically in absence of an immune response, (b) both natural killer and CD8^+^T cells are capable of killing tumor cells, (c) both natural killers and CD8^+^T cells respond to tumor cells by expanding and increasing cytolytic activity, (d) natural killers are normally present in the body, even when no tumor cells are present, (e) active tumor specific CD8^+^T cells are only present in large numbers when tumor cells are present, and (f) both CD8^+^T cells and natural killers become inactive after some number of encounters with tumor cells [40, 42]. Arabameri et al. [8] proposed a model for immunotherapy using mature dendritic cells. Mamat et al. [2] proposed a modified model that includes the effect of both IL-2 and INF-*α*. A model was proposed by Eftimie et al. [44] and was modified by Anderson et al. [45] and by Hu and Jang [46] to study the effect of CD4^+^T cells on the system. CD4^+^T cells are helper cells that activate CD8^+^T cells to achieve a regulated immune response and protect against tumors. However, CD4^+^T cells can play a direct role in killing tumor cells via secretion of cytokines in addition to its traditional helper role [45–49].

Intriguingly, previous studies modelled the effect of natural killer cells, circulating lymphocytes and CD8^+^T cells, and neglected the role of CD4^+^T cells, or studied the role of CD4^+^T cells, neglecting the role of other immune cells. In this study, a mathematical model is proposed for active immunotherapy that includes the effects of natural killer cells, circulating lymphocytes, CD8^+^T cells, CD4^+^T cells, and chemotherapy on the tumor. Both roles of CD4^+^T cells are considered in the model. The work of de Pillis et al. [13, 40–42], Eftimie et al. [44], Anderson et al. [45], and Hu and Jang [46] are the main references for the proposed model. The model is solved numerically using the “Adams predictor-corrector” method with Adams–Bashforth predictor and Adams–Moulton corrector. A stability analysis is performed for patients' data to determine if the system is stable. For a nonzero tumor case, the tumor is totally eradicated by the treatment intervention and the possibility of recurrence is to be studied. At the zero tumor case, the system must be stable to avoid recurrence and a condition for stability is deduced. It is recommended to work on modifying the patient's body parameters, using cancer vaccines, for example, before applying the treatment intervention in order to satisfy the condition needed for stability [42]. Finally, bifurcation analysis is performed to study the effect of changing patient's parameters on stability.

## 2. Materials and Methods

### 2.1. Mathematical Model

To illustrate the interaction between tumor cells *T*(*t*) and immune cells present in the microenvironment of the tumor without external effects, such as treatment, the following model is proposed:

(1) $$\begin{array}{c} \frac{\text{d}T}{\text{d}t}=a\;T\;\left(1-b\;T\right)-c\;N\;T-D\;T-\frac{c_{1}\;T}{a_{1}+\;T}\;I, \end{array}$$

(2) $$\begin{array}{c} \frac{\text{d}N}{\text{d}t}=e\;C-f\;N+\frac{g\;T^{2}}{h+\;T^{2}}\;N-p\;N\;T, \end{array}$$

(3) $$\begin{array}{c} \frac{\text{d}L}{\text{d}t}=-m\;L+\frac{j\;D^{2}\;T^{2}}{k+\;D^{2}\;T^{2}}\;L-q\;L\;T+\left(r_{1}\;N+r_{2}\;C\right)\;T-u\;N\;L^{2}+\frac{p_{i}\;I}{g_{i}+I}L, \end{array}$$

(4) $$\begin{array}{c} \frac{\text{d}Y}{\text{d}t}=\frac{\beta_{1}\;T}{\alpha_{1}+\;T}\;I-\mu_{1}\;Y-\delta_{2}\;T\;Y, \end{array}$$

(5) $$\begin{array}{c} \frac{\text{d}C}{\text{d}t}=\alpha -\beta \;C, \end{array}$$

(6) $$\begin{array}{c} \frac{\text{d}I}{\text{d}t}=-\mu_{i}I+\frac{\beta_{2}\;T}{\alpha_{2}+\;T}\;Y, \end{array}$$

where *N*(*t*) is the natural killer cell population, *L*(*t*) is the CD8^+^T cell population, *Y*(*t*) is the CD4^+^T cell population,  *C*(*t*) is the circulating lymphocyte cell population not including natural killer cells and active CD8^+^T and CD4^+^T cells [50, 51], *I*(*t*) is the concentration of the IL-2 cytokine in the body, and *D* is defined as

(7) $$\begin{array}{c} D=d\frac{{\left(L/T\right)}^{l}}{s+\;{\left(L/T\right)}^{l}}\;, \end{array}$$

and the parameters *a*,  *a*_1_, *b*, *c*, *c*_1_,  *d*, *e*, *f*, *g*,  *g*_*i*_, *h*, *j*, *k*,  *l*,  *m*, *p*,  *p*_*i*_, *q*, *r*_1_, *r*_2_, *s*, *u*, *α*,  *α*_1_, *α*_2_, *β*, *β*_1_, *β*_2_, *μ*_1_, *μ*_*i*_, and *δ*_2_ are given in Table 1. The role of CD4^+^T cells is included in this model, and it depends mainly upon the work of Eftimie et al. [44], Anderson et al. [45], and Hu and Jang [46]. In this model, CD4^+^T is included in equation (4), and its effect is included in the terms (*c*_1_ *T*/(*a*_1_ +  *T*)) *I* and (*β*_2_ *T*/(*α*_2_ +  *T*)) *Y* in equations (1) and (6) [46, 54]. In equation (1), *a* *T* (1 − *b* *T*) represents tumor growth and is assumed to be logistic [40, 42, 46]. The term −*cNT* represents the natural killer-induced tumor death [40, 42]. The next term −*D* *T* is the tumor lysis by CD8^+^T cells [40, 42]. Unlike CD8^+^T cells, CD4^+^T cells cannot kill tumor cells directly but through the cytokines that they produce. The role of cytokine in killing the tumor is represented by the term –(*c*_1_ *T*/(*a*_1_ +  *T*)) *I* in equation (1) [46, 54]. In equation (2), *eC* represents the production of natural killer cells from circulating lymphocytes [40, 42]. This growth term is tied to the overall immune health levels as measured by the population of circulating lymphocytes [54–56]. −*fN* represents natural killers' turnover [51]. The term (*g* *T*^2^/(*h* +  *T*^2^)) *N* represents the recruitment of natural killer cells induced due to the existence of the tumor [42, 54, 57]. The next term −*p* *N* *T* is natural killer death by exhaustion of tumor-killing resources [40, 42, 51]. In equation (3), −*m* *L* represents the inactivation of CD8^+^T cells consisting only of natural death rate since no CD8^+^T cells are assumed to be present in the absence of tumor cells [40, 42]. The term (*j* *D*^2^ *T*^2^/(*k* + *D*^2^ *T*^2^))*L* represents the recruitment in CD8^+^T cell population due to the presence of the tumor [40, 42, 58]. The next term −*qLT* is the CD8^+^T cell death by exhaustion of tumor-killing resources [40, 42, 51]. The term *r*_1_*NT* represents CD8^+^T cell stimulation by natural killer-lysed tumor cell debris, and the term *r*_2_*CT* represents the activation of native CD8^+^T cells in the general lymphocyte population [42, 51]. The term −*u* *N* *L*^2^ is the natural killer cell regulation of CD8^+^T cells. It describes regulation and suppression of CD8^+^T cell activity, which occurs when there are very high levels of activated CD8^+^T cells without responsiveness to cytokines present in the system [40, 42, 59, 60]. The activating effect of cytokines on CD8^+^T cells is represented in equation (3) by the term (*p*_*i*_ *I*/(*g*_*i*_ + *I*))*L* [40, 42, 61].

CD4^+^T cell population is represented by equation (4) which has a constant decaying rate *μ*_1_. The other decaying term −*δ*_2_ *T* *Y* exists due to the interaction with the tumor. Activating effect of cytokines on CD4^+^T cells due to existence of the tumor is represented by the term (*β*_1_ *T*/(*α*_1_ +  *T*)) *I* in equation (4) [46, 54]. Equation (5) represents the circulating lymphocytes cell population which are assumed to be generated at a constant rate *α* and death rate *β* [40, 42, 54–56]. Equation (6) represents the concentration of cytokines which is decaying with a constant rate *μ*_*i*_. The activating role of the CD4^+^T cells on cytokines due to the existence of the tumor is represented by the term ((*β*_2_ *T*)/(*α*_2_ +  *T*)) *Y* in equation (6) [46, 54].

The previous model represents the interaction of patient's body with the tumor only without any treatment. It can be generalized to introduce the effects of chemotherapy and/or immunotherapy; refer, for example, de Pillis and Radunskaya [40] and Hu and Jang [46]. The general model takes the following form:

(8) $$\begin{array}{c} \frac{\text{d}T}{\text{d}t}=a\;T\;\left(1-b\;T\right)-cNT-D\;T-\frac{c_{1}T}{a_{1}+\;T}\;I-K_{T}\left(1-e^{-M}\right)\;T, \end{array}$$

(9) $$\begin{array}{c} \frac{\text{d}N}{\text{d}t}=eC-fN+\frac{gT^{2}}{h+\;T^{2}}\;N-pNT-K_{N}\left(1-e^{-M}\right)\;N, \end{array}$$

(10) $$\begin{array}{c} \frac{\text{d}L}{\text{d}t}=-m\;L+\frac{j\;D^{2}\;T^{2}}{k+\;D^{2}\;T^{2}}\;L-q\;L\;T+\left(r_{1}\;N+r_{2}\;C\right)\;T-u\;N\;L^{2}+\frac{p_{i}\;I}{g_{i}+I}L-K_{L}\left(1-e^{-M}\right)\;L+v_{L}\left(t\right), \end{array}$$

(11) $$\begin{array}{c} \frac{\text{d}Y}{\text{d}t}=\frac{\beta_{1}\;T}{\alpha_{1}+\;T}\;I-\mu_{1}\;Y-\delta_{2}T\;Y+v_{Y}\left(t\right), \end{array}$$

(12) $$\begin{array}{c} \frac{\text{d}C}{\text{d}t}=\alpha -\beta \;C-K_{C}\left(1-e^{-M}\right)\;C, \end{array}$$

(13) $$\begin{array}{c} \frac{\text{d}I}{\text{d}t}=-\mu_{i}I+\frac{\beta_{2}\;T}{\alpha_{2}+\;T}\;Y+v_{I}\left(t\right), \end{array}$$

(14) $$\begin{array}{c} \frac{\text{d}M}{\text{d}t}=-\gamma \;M+v_{M}\left(t\right), \end{array}$$

such that *v*_*M*_(*t*), *v*_*I*_(*t*), *v*_*L*_(*t*), and *v*_*Y*_(*t*) are the concentrations of chemotherapy, IL-2 cytokine therapy, CD8^+^T, and CD4^+^T adoptive immunotherapy, respectively. The effect of chemotherapy on immune cells is represented by the terms −*K*_*N*_(1 − *e*^−*M*^) *N*, −*K*_*L*_(1 − *e*^−*M*^) *L*, and −*K*_*C*_(1 − *e*^−*M*^) *C*. The three terms illustrate the reduction in number of each type of immune cell populations, while tumor cell killing due to chemotherapy is represented by −*K*_*T*_(1 − *e*^−*M*^) *T* and the concentration of the chemotherapeutic agent is represented by equation (14) [40, 42]. The parameters *K*_*T*_,  *K*_*N*_,  *K*_*L*_,  *K*_*C*_, and *γ* are defined in Table 1.

### 2.2. Method of Solution

In numerical methods for solving differential equations, a problem may occur when small changes in the independent variable lead to large changes in the dependent variable. If this problem exists in a system of differential equations, the system is called a stiff system [62, 63]. The treatment doses *v*_*L*_(*t*),  *v*_*Y*_(*t*),  *v*_*I*_(*t*), and *v*_*M*_(*t*) change suddenly from zero to very large values within small intervals of  *t*, and systems (8)–(14) become a stiff system. We tried to solve the system using the Runge–Kutta method, but the solution was divergent in most cases due to the stiffness. The Adams–Bashforth method [62–65] is a powerful method for solving stiff problems and was originally proposed in 1883. For the following nonlinear initial value problem,

(15) $$\begin{array}{c} y\prime \left(t\right)=f\left(t,y\right),\text{ where }y\left(t_{0}\right)=y_{0}. \end{array}$$

The approximate solution at *t*_*n*_ is given by

(16) $$\begin{array}{c} y_{n}=y_{n-1}+\Delta t\;\left(\overset{k}{\underset{i=1}{\sum }}\beta_{i}\;f\left(t_{n-i},y_{n-i}\right)\right), \end{array}$$

where Δ*t* is the step size and *k* is the order of the method. The coefficients *β*_1_, *β*_2_,… , *β*_*k*_  can be deduced using Taylor's expansion of *y*(*t*) near the point *t*=*t*_0_ with error of order *O*((Δ*t*)^*k*+1^) [65]. This means that, at any point, we can find the solution numerically using any number of the previous points.

The Adams–Moulton method [62, 63, 65, 66] is a modification of the Adams–Bashforth method, which was proposed in 1926 by adding an implicit term to equation (16) to become

(17) $$\begin{array}{c} y_{n}=y_{n-1}+\Delta t\;\left(\beta_{0}\;f\left(t_{n},y_{n}\right)+\overset{k}{\underset{i=1}{\sum }}\beta_{i}\;f\left(t_{n-i},y_{n-i}\right)\right). \end{array}$$

Coefficients can be found in the same way of Adams–Bashforth method.

Finally, a combined two-staged method is proposed by first computing the predictor stage using the Adams–Bashforth method [62, 65, 67]:

(18) $$\begin{array}{c} y_{n}^{*}=y_{n-1}+\Delta t\;\left(\overset{k}{\underset{i=1}{\sum }}\beta_{i}^{*}\;f\left(t_{n-i},y_{n-i}\right)\right), \end{array}$$

and then the corrector stage using the Adams–Moulton formula:

(19) $$\begin{array}{c} y_{n}=y_{n-1}+\Delta t\;\left(\beta_{0}\;f\left(t_{n},y_{n}^{*}\right)+\overset{k}{\underset{i=1}{\sum }}\beta_{i}\;f\left(t_{n-i},y_{n-i}\right)\right). \end{array}$$

This combined method is called the “Adams predictor-corrector method.” In this paper, we use it to solve the systems of nonlinear ordinary differential equations presented by equations (1)–(6) and (8)–(14).

## 3. Results and Discussion

The model proposed in this study is solved numerically using the Adams predictor-corrector method up to the 12^th^ order, and the solutions are interpolated. The method has nonzero stability ordinates for this order [68]. Values of the parameters *a*,  *a*_1_, *b*, *c*, *c*_1_, *d*, *e*, *f*, *g*,  *g*_*i*_, *h*, *j*, *k*, *K*_*T*_,  *K*_*N*_,  *K*_*L*_,  *K*_*C*_,  *l*,  *m*, *p*,  *p*_*i*_, *q*, *r*_1_, *r*_2_, *s*,  *u*, *α*,  *α*_1_, *α*_2_, *β*,  *β*_1_, *β*_2_, *γ*,  *μ*_1_,  *μ*_*i*_, and *δ*_2_ are given in Table 1 for humans. The solutions are obtained for three cases: without any treatment, with continuous treatment, and with pulsed treatment as detailed below.

### 3.1. First: A Case of No Treatment

**Figure 1:**
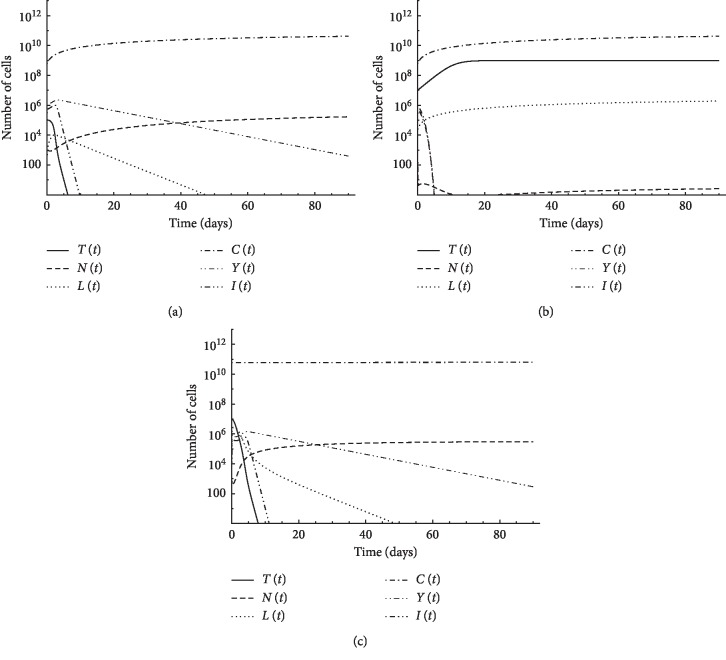
Simulation of no-treatment case for first patient's data: (a) initial tumor size *T* (0) = 10^5^; (b) initial tumor size *T* (0) = 10^7^; (c) initial tumor size *T* (0) = 10^7^ and immune cells: *L* (0) = 10^2^ and *C* (0) = 6 × 10^10^.

In this case, we illustrate the interaction between tumor cells and the immune system without any treatment. In Figures 1(a) and 1(b), simulation was performed for first patient's data where the initial natural killer cells *N*(0)=10^3^, initial CD8^+^T cells *L*(0)=10, initial CD4^+^T cells *Y*(0)=10^6^, and initial circulating lymphocytes *C*(0)=6 × 10^8^. The immune system could overcome tumor of size *T*(0)=10^5^, as shown in Figure 1(a), but cannot overcome a tumor of size *T*(0)=10^7^, as shown in Figure 1(b). The simulation is performed again with tumor size *T*(0)=10^7^ but with a stronger immune system such that *N*(0)=10^3^, *L*(0)=10^2^, *Y*(0)=10^6^, and *C*(0)=6 × 10^10^, which means that initial CD8^+^T cells and initial circulating lymphocytes are increased.  Figure 1(c) shows that tumor cells were destroyed in such case.

In this section, we demonstrate the role of different immune cells in abolishing the tumor. The results suggest that patient's body can overcome cancer cells up to a certain size. In case of larger tumors, however, a stronger immune system is necessary to overcome cancer cells.

### 3.2. Second: A Case of Continuous Treatment

**Figure 2:**
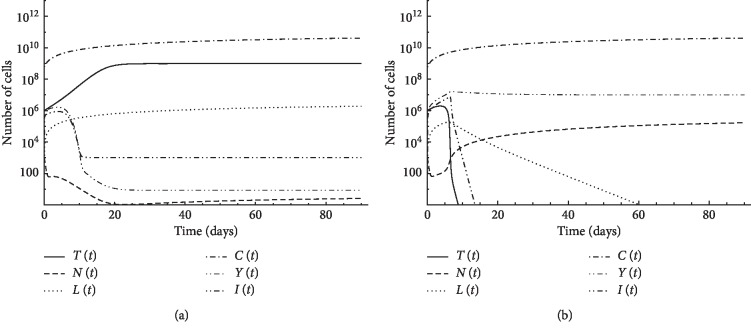
Simulation of continuous treatment case for first patient's data: (a) continuous CD8^+^T cells and IL-2 treatments; (b) continuous CD4^+^T cell treatments.

In this case, we simulated the model with continuous immunotherapy. This is performed using the first patient's data, with initial natural killer cells *N*(0)=10^3^, initial CD8^+^T cells *L*(0)=10, initial CD4^+^T cells *Y*(0)=10^6^, initial circulating lymphocytes *C*(0)=6 × 10^8^, and initial tumor size *T*(0)=10^6^. First, a continuous adoptive therapy is introduced using CD8^+^T cells with concentration *v*_*L*_=10^4^ and cytokine IL-2 with concentration *v*_*I*_=10^4^.  Figure 2(a) demonstrates that the tumor cannot be eliminated using this treatment. Second, a continuous adoptive therapy is introduced using CD4^+^T cells with concentration *v*_*Y*_=10^6^ instead of both CD8^+^T cells and IL-2 cytokine therapies, and the tumor could be eliminated, as shown in Figure 2(b).

**Figure 3:**
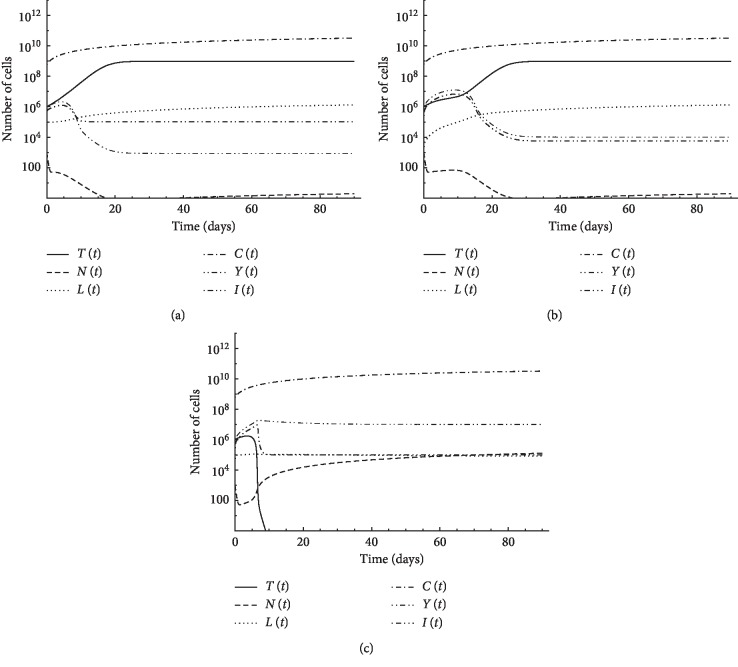
Simulation of continuous treatment case for second patient's data: (a) continuous CD8^+^T cells and IL-2 treatments; (b) continuous CD4^+^T cells treatments; (c) continuous CD8^+^T cells, IL-2, and CD4^+^T cell treatments.

Treatment is applied using the second patient's data with initial conditions: *N*(0)=10^3^, *L*(0)=10^2^, *Y*(0)=10^6^, *C*(0)=6 × 10^8^, and tumor size *T*(0)=10^6^.  Figure 3(a) illustrates the results of using continuous CD8^+^T cell therapy *v*_*L*_=10^6^ together with cytokine IL-2 treatment *v*_*I*_=10^6^, and Figure 3(b) shows the result when using only CD4^+^T cell therapy *v*_*Y*_=10^6^. In both cases, the tumor is not eliminated, but when using the three therapies together, *v*_*L*_=10^6^, *v*_*I*_=10^6^, and *v*_Y_=10^6^, the tumor is eliminated in about 10 days, as shown in Figure 3(c).

### 3.3. Third: A Case of Pulsed Treatment

**Figure 4:**
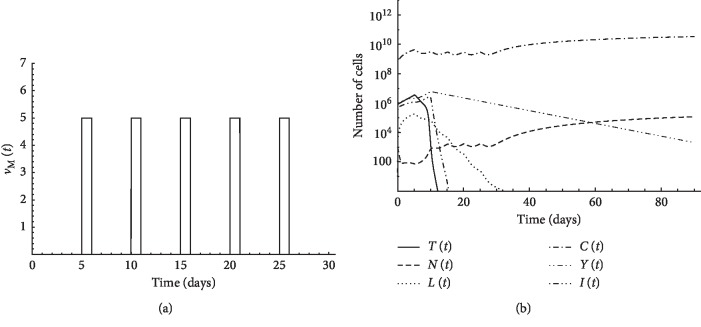
Simulation of pulsed chemotherapy for first patient's data: (a) 5 pulses of chemotherapy *v*_*M*_ = 5 with 5 days period starting from day 6; (b) initial tumor size *T* (0) = 8 × 10^5^.

**Figure 5:**
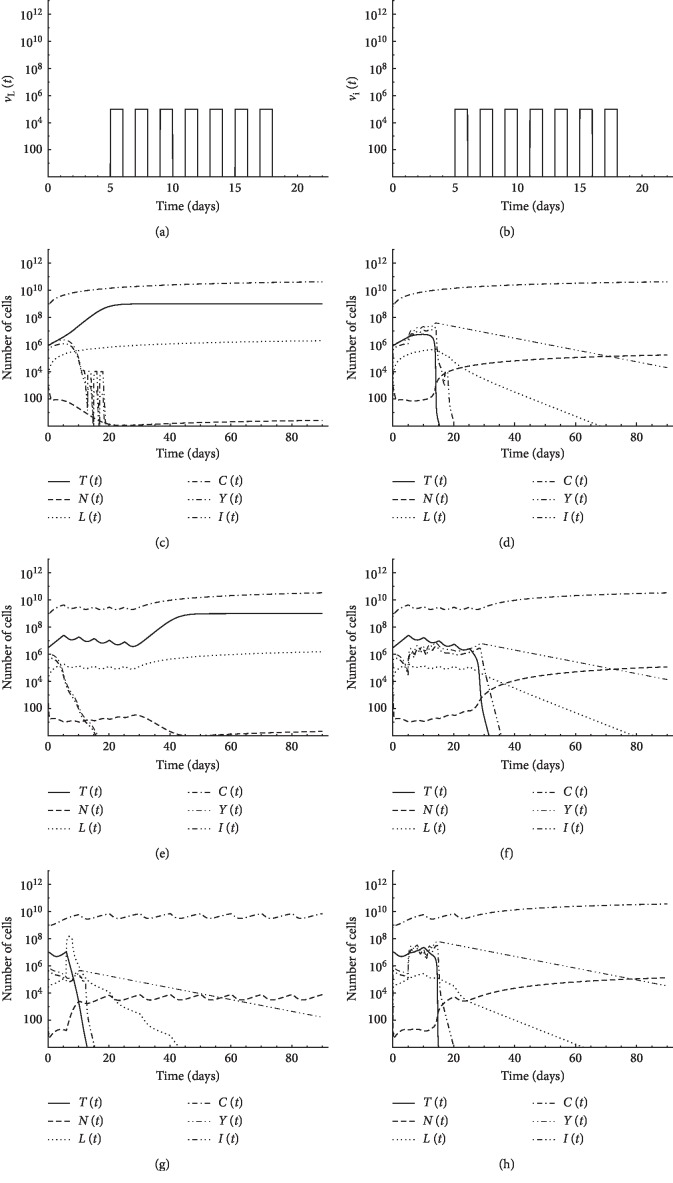
Simulation of pulsed treatment case for first patient's data: (a) 7 pulses of CD8^+^T cell treatment *v*_*L*_ = 10^5^ with 2 days period starting from day 6; (b) 7 pulses of IL-2 cytokine *v*_*I*_ = 10^5^ with 2 days period starting from day 6; (c) pulsed CD8^+^T cells and IL-2 treatment with initial tumor size *T*(0) = 8 × 10^5^; (d) pulsed CD8^+^T cells and IL-2 combined with CD4^+^T cell treatments with initial tumor size *T*(0) = 8×10^5^; (e) pulsed chemotherapy with initial tumor size *T*(0) = 3 × 10^6^; (f) pulsed chemotherapy combined with CD4^+^T cell treatment with initial tumor size *T*(0) = 3 × 10^6^; (g) pulsed chemotherapy combined with CD8^+^T cells and IL-2 treatments with initial tumor size *T*(0) = 10^7^; (h) pulsed chemotherapy combined with CD4^+^T cell treatment with initial tumor size *T*(0) = 10^7^.

In this case, the patient is administered therapy on certain days only. In Figures 4(b), 5(c), and 5(d), a tumor of initial size *T*(0)=8 × 10^5^ is assumed to exist in the first patient with initial conditions: *N*(0)=10^3^, *L*(0)=10^2^, *Y*(0)=10^6^, and *C*(0)=6 × 10^8^. In each figure, we use a certain regimen of treatment. In Figure 4(a), five pulses of chemotherapy of intensity *v*_*M*_=5 starting from the 6^th^ day with 5 days period can eliminate the tumor, as shown in Figure 4(b). An alternative to chemotherapy, 7 pulses of CD8^+^T cell treatment *v*_*L*_=10^5^ combined with IL-2 cytokine *v*_I_=10^5^ with 2 days period starting from day 6 were proposed, as illustrated in Figures 5(a) and 5(b).  Figure 5(c) demonstrates that this combination is not enough to eradicate the tumor. Oscillations of CD4^+^T cell population and IL-2 cytokine concentration are experienced due to fast consumption of the treatment by the resisting tumor cells. These large changes in small time intervals explain the reason of the failure of the Runge–Kutta method in obtaining the numerical solution. When we use the same treatment schedule with additional 5 pulses of CD4^+^T cell treatment *v*_*Y*_=10^7^ with 2 days period starting from day 6, the tumor is eliminated, as shown in Figure 5(d). These simulations suggest that CD4^+^T cells could replace chemotherapy and thus could spare patients the agony of chemotherapy-induced toxicities and side effects.

Pulsed treatment is applied to the first patient with initial conditions: *N*(0)=10^3^, *L*(0)=10, *Y*(0)=10^6^, and *C*(0)=6 × 10^8^ but with a larger tumor size *T*(0)=3 × 10^6^. In Figure 5(e), five pulses of chemotherapy of intensity *v*_*M*_=5 is applied starting from the 6^th^ day with 5 days period. Chemotherapy alone is insufficient to overcome tumor growth. In Figure 5(f), the same chemotherapy drug is applied combined with 5 pulses of CD4^+^T cell therapy *v*_Y_=6 × 10^6^ with 2 days period starting from day 6. Combined treatment could abolish the tumor, while chemotherapy alone was not able to do so. The results suggest that, depending on tumor size, CD4^+^T cell treatment can be an alternative to chemotherapy or is needed in combination with therapy.

De Pillis and Radunskaya [40] studied the case of a larger tumor of initial size *T*(0)=10^7^. Pulsed treatment is applied to the first patient with initial conditions: *N*(0)=10^3^, *L*(0)=10, and *C*(0)=6 × 10^8^. They applied 9 pulses of chemotherapy of intensity *v*_*M*_=5 starting from the 1^st^ day with 10 days period combined with 6 pulses of the IL-2 cytokine therapy *v*_*I*_=5 × 10^5^ from day 8 to day 11 and CD8^+^T cells treatment *v*_L_=10^9^ during days 7 and 8. Combined treatment could abolish the tumor in this case, as shown in Figure 5(g). As an alternative to this therapy, with the same initial conditions, however, we added the effect of CD4^+^T cells (*Y*(0)=10^6^). We suggest 5 pulses of CD4^+^T cell therapy *v*_*Y*_=4 × 10^7^ with 2 days period starting from day 6 combined with three pulses of chemotherapy of intensity *v*_*M*_=5 applied starting from the 1^st^ day with 10 days period.  Figure 5(h) illustrates that, in this case, the tumor can be also abolished. It is observed that we used a lower dose of the chemotherapeutic agent. Also, we used CD4^+^T cell therapy as an alternative to both CD8^+^T cell therapy and cytokine therapy.

### 3.4. Stability Analysis

In this section, stability analysis is performed for the system of equations (1) to (6). The main advantage of studying stability is to investigate the possibility of eradicating the tumor without the occurrence of recurrence. If an equilibrium point is stable, it will attract the system towards it, in its domain of attraction. However, if the equilibrium point is unstable, any small perturbation from equilibrium will cause the system to move away from this equilibrium point; i.e., upon terminating treatment, the tumor will escape immune surveillance unless every single tumor cell is killed. To avoid recurrence of tumor after treatment, the zero tumor case has to be stable. In this section, a condition for stability of the zero tumor case is deduced. The first step is equating the right-hand sides of these unforced autonomous equations to zero [69–72]. From equation (1), we can deduce that either *T* = 0 or *a* (1 − *b* *T*) − *c* *N* − *D* − (*c*_1_/(*a*_1_ +  *T*)) *I* = 0 is at equilibrium. In the following part, the two equilibrium conditions are considered; first, the zero tumor equilibrium, *T*_eq_ = 0, and then the nonzero tumor equilibrium, *a* (1 − *b* *T*_eq_) − *c* *N*_eq_ − *D*_eq_ − (*c*_1_/(*a*_1_ +  *T*_eq_)) *I*_eq_ = 0 and *T*_eq_ ≠ 0.

#### 3.4.1. Equilibrium and Stability for Zero Tumor

In this case, *T*_eq_=0. Substituting in equations (4)–(6), the zero tumor equilibrium yields *Y*_eq_=0, *C*_eq_=*α*/*β*, and *I*_eq_=0. By substituting in equation (2) at equilibrium, it follows that *N*_eq_=(*eα*)/(*fβ*). Finally, substituting in equation (3) at equilibrium, the result is −*m* *L*_eq_ − *u* *N*_eq_ *L*_eq_^2^=0, then  *L*_*eq*_=0, and the negative solution for *L*_eq_ is refused.

The zero tumor equilibrium point is

(20) $$\begin{array}{c} \left(\;T_{\text{eq}},N_{\text{eq}},L_{\text{eq}},Y_{\text{eq}},C_{\text{eq}},I_{\text{eq}}\;\right)=\left(\;0\;,\frac{e\alpha }{f\beta }\;,0\;,0\;,\frac{\alpha }{\beta }\;,0\;\right)\;. \end{array}$$

Linearizing system of equations (1) to (6) near the zero tumor equilibrium point, then

(21) $$\begin{array}{c} \frac{\text{d}T}{\text{d}t}=\left(a-\frac{ce\alpha }{f\beta }\right)\;T+\text{H}.\text{O}.\text{T}.,\\ \frac{\text{d}N}{\text{d}t}=-f\;\left(N-\frac{e\;\alpha }{f\;\beta }\right)+e\;\left(C-\frac{\alpha }{\beta }\right)+\text{H}.\text{O}.\text{T}.,\\ \frac{\text{d}L}{\text{d}t}=\left(r_{1}\;\frac{e\;\alpha }{f\;\beta }+r_{2}\;\frac{\alpha }{\beta }\right)\;T-m\;L+\text{H}.\text{O}.\text{T}.,\\ \frac{\text{d}Y}{\text{d}t}=-\mu_{1}\;Y+\text{H}.\text{O}.\text{T}.,\\ \frac{\text{d}C}{\text{d}t}=\alpha -\beta \;C,\\ \frac{\text{d}I}{\text{d}t}=-\mu_{i}I+\text{H}.\text{O}.\text{T}.\;. \end{array}$$

The Jacobian matrix [69–71] of this system is

(22) $$\begin{array}{c} J=\left[\begin{array}{cccccc} a-\left(\frac{c\;e\;\alpha }{f\;\beta }\right) & 0 & 0 & 0 & 0 & 0\\ 0 & -f & 0 & 0 & e & 0\\ r_{1}\left(\frac{e\;\alpha }{f\;\beta }\right)+r_{2}\left(\frac{\alpha }{\beta }\right) & 0 & -m & 0 & 0 & 0\\ 0 & 0 & 0 & -\mu_{1} & 0 & 0\\ 0 & 0 & 0 & 0 & -\beta & 0\\ 0 & 0 & 0 & 0 & 0 & -\mu_{i} \end{array}\right]. \end{array}$$

The system has the eigenvalues *λ*_1_ = (*a* − (*c* *e* *α*/(*f* *β*))), *λ*_2_ = −*f*, *λ*_3_ = −*m*, *λ*_4_ = −*μ*_1_, *λ*_5_ = −*β*, and *λ*_6_ = −*μ*_*i*_. All the eigenvalues are always negative except the first eigenvalue which is negative only when *a* − (*ceα*)/(*fβ*) < 0. Then, the tumor-free state is a stable state if *a* − (*ceα*)/(*fβ*) < 0 and unstable otherwise.

#### 3.4.2. Equilibrium and Stability for Nonzero Tumor

In this case,

(23) $$\begin{array}{c} a\left(1-b\;T_{\text{eq}}\right)-cN_{\text{eq}}-D_{\text{eq}}-\frac{c_{1}\;}{a_{1}+\;T_{\text{eq}}}\;I_{\text{eq}}=0, \end{array}$$

then,

(24) $$\begin{array}{c} D_{\text{eq}}=a\left(1-b\;T_{\text{eq}}\right)-cN_{\text{eq}}-\frac{c_{1}\;}{a_{1}+\;T_{\text{eq}}}\;I_{\text{eq}}. \end{array}$$

From equations (2) and (5) at equilibrium,

(25) $$\begin{array}{c} N_{\text{eq}}=\frac{e\;\alpha \;\left(h+\;T_{\text{eq}}^{2}\right)}{\beta \left(pT_{\text{eq}}^{3}+\left(f-g\right)\;T_{\text{eq}}^{2}+p\;h\;T_{\text{eq}}+f\;h\right)}. \end{array}$$

From equation (7) at equilibrium,

(26) $$\begin{array}{c} L_{\text{eq}}={\left(\frac{s\;D_{\text{eq}}}{d-D_{\text{eq}}}\right)}^{1/l}T_{\text{eq}}. \end{array}$$

From equation (3) at equilibrium,

(27) $$\begin{array}{c} uN_{\text{eq}}L_{\text{eq}}^{2}+\left(m-\frac{j\;D_{\text{eq}}^{2}\;T_{\text{eq}}^{2}}{k+\;D_{\text{eq}}^{2}\;T_{\text{eq}}^{2}}+q\;T_{\text{eq}}-\frac{p_{i}I_{\text{eq}}}{g_{i}+I_{\text{eq}}}\right)L_{\text{eq}}-\left(r_{1}\;N_{\text{eq}}+r_{2}\frac{\alpha }{\beta }\right)\;T_{\text{eq}}=0. \end{array}$$

From equations (4) and (6) at equilibrium,

(28) $$\begin{array}{c} \left(\;\frac{\beta_{1}\;T_{\text{eq}}}{\left(\alpha_{1}+\;T_{\text{eq}}\right)}-\frac{\mu_{i}\left(\alpha_{2}+\;T_{\text{eq}}\right)\left(\mu_{1}+\delta_{2}\;T_{\text{eq}}\right)}{\beta_{2}\;T_{\text{eq}}}\right)\;I_{\text{eq}}=0. \end{array}$$

Then, from equation (28), the nonzero tumor equilibrium will be divided into two cases: *the zero cytokine case* at which *I*_*eq*_ = 0 and *nonzero cytokine case* at which ((*β*_1_ *T*_eq_/(*α*_1_ +  *T*_eq_)) − (*μ*_*i*_(*α*_2_ +  *T*_eq_)(*μ*_1_ + *δ*_2_ *T*_eq_)/(*β*_2_ *T*_eq_))) = 0 and *I*_eq_ ≠ 0. In the nonzero tumor equilibrium with zero cytokine, substitution in equation (4) at equilibrium gives *Y*_eq_ = 0. Equilibrium points in this case can be found by solving equations (26) and (27) for *T*_eq_ and *L*_eq_ and then calculating other variables. Data for the two patients are used for numerical solution. Since equations (26) and (27) are highly nonlinear, the solution is obtained by plotting both of them in the *T* − *L* plane and determining the intersections. In the nonzero tumor equilibrium with nonzero cytokine,

(29) $$\begin{array}{c} \left(\;\frac{\beta_{1}\;T_{\text{eq}}}{\left(\alpha_{1}+\;T_{\text{eq}}\right)}-\frac{\mu_{i}\left(\alpha_{2}+\;T_{\text{eq}}\right)\left(\mu_{1}+\delta_{2}\;T_{\text{eq}}\right)}{\beta_{2}\;T_{\text{eq}}}\right)=0. \end{array}$$

Rearranging,

(30) $$\begin{array}{c} T_{\text{eq}}^{3}+\left(\alpha_{1}+\alpha_{2}+\frac{\mu_{1}}{\delta_{2}}-\frac{\beta_{1}\beta_{2}}{\delta_{2}\mu_{i}}\right)T_{\text{eq}}^{2}+\left(\alpha_{1}\alpha_{2}+\frac{\mu_{1}\alpha_{2}+\mu_{1}\alpha_{1}}{\delta_{2}}\right)T_{\text{eq}}+\frac{\mu_{1}\alpha_{1}\alpha_{2}}{\delta_{2}}=0. \end{array}$$

The equilibrium points in this case can be found by solving equation (30) for *T*_eq_. For each value of *T*_eq_, equations (26) and (27) are solved for *I*_eq_ and *L*_eq_ and then substituted for other variables. Due to the high nonlinearity of the two equations, the solution is obtained by plotting both of them in the *I* − *L* plane and determining the intersections. For each equilibrium point, a linearization method is used to write the Jacobian and to determine the eigenvalues. Consequently, the stability of the system is specified.

#### 3.4.3. Results of Stability Analysis for Both the Zero Tumor and Nonzero Tumor

**Figure 6:**
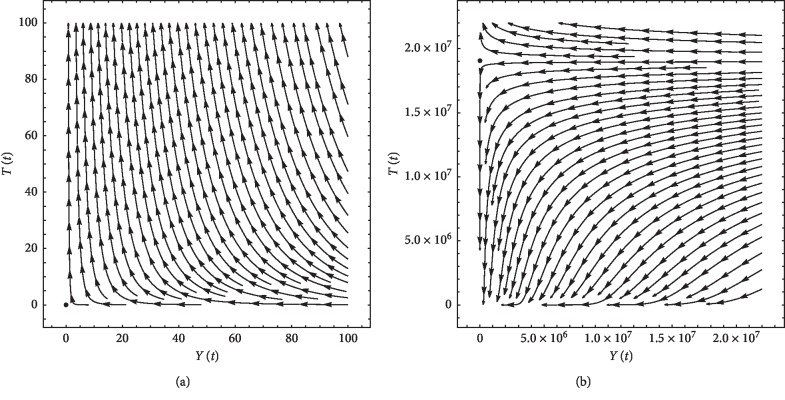
Streamlines for first patient's data: (a) at equilibrium point 1, *N*_eq_ = 315534, *L*_eq_ = 0, *C*_eq_ = 6.25 × 10^10^, and *I*_eq_ = 0; (b) at equilibrium point 2, *N*_eq_ = 199.335, *L*_eq_ = 2.82461 × 10^6^, *C*_eq_ = 6.25 × 10^10^, and *I*_eq_ = 0.

Table 2 illustrates the results for the parameter values given in Table 1. For more illustration, streamlines are plotted. We use the values of *N*_eq_, *L*_eq_, *C*_eq_, and *I*_eq_ of each equilibrium point and allow *Y* and *T* to vary with time according to equations (1) and (4). For the first patient, the streamlines are plotted for equilibrium point 1, *T* = 0, and *Y* = 0, in Figure 6(a). One can notice that, for any state of the system in the vicinity of this unstable equilibrium point, the system moves away from that point. The streamlines are plotted for equilibrium point 2, *T* = 1.90609 × 10^7^ and *Y* = 0, in Figure 6(b). Also, for this unstable equilibrium point, any state of the system in the vicinity of this point, the system moves away from it. In the same manner, the streamlines for the second patient are obtained; however, to be concise, they are not included.

In this analysis, it is shown that all equilibrium points are unstable for both patients. This means that, for zero tumor case, the tumor can recur, and in nonzero tumor case, the size of the tumor is uncontrollable; i.e., it could increase without a limit. For the zero tumor, an analytical expression for the stability of the equilibrium point is obtained. Simply, if the condition *a* − (*ceα*)/(*fβ*) < 0 is satisfied, then the zero tumor case is a stable state and the tumor cannot recur. If the condition is not satisfied, then the tumor-free state is an unstable state and the tumor is recurrent. Though for the nonzero tumor the stability analysis is analysed numerically, changing the system parameters could lead to stable equilibrium. This is tackled in the next section. Hence, it is recommended for any successful treatment to work on modifying the patient's body parameters in order to satisfy the stability condition.

### 3.5. Bifurcation Analysis

A bifurcation analysis is performed to study the effect of changing patient's parameters on the system stability [15, 40, 42, 73]. This change could lead to turning unstable point(s) into stable; hence, for nonzero tumor, the size is limited and can be reduced by surgery or radiation and then immunotherapy and/or chemotherapy to reach a stable zero tumor case. In the zero tumor case, it is required to change patient's parameters, using cancer vaccines, for example, to satisfy the condition of stability stated in the previous section and prevent recurrence. Bifurcation diagram is plotted for parameters *a* and *c* for the first patient and the second patient. In the same manner, bifurcation analysis can be studied for other system parameters.

**Figure 7:**
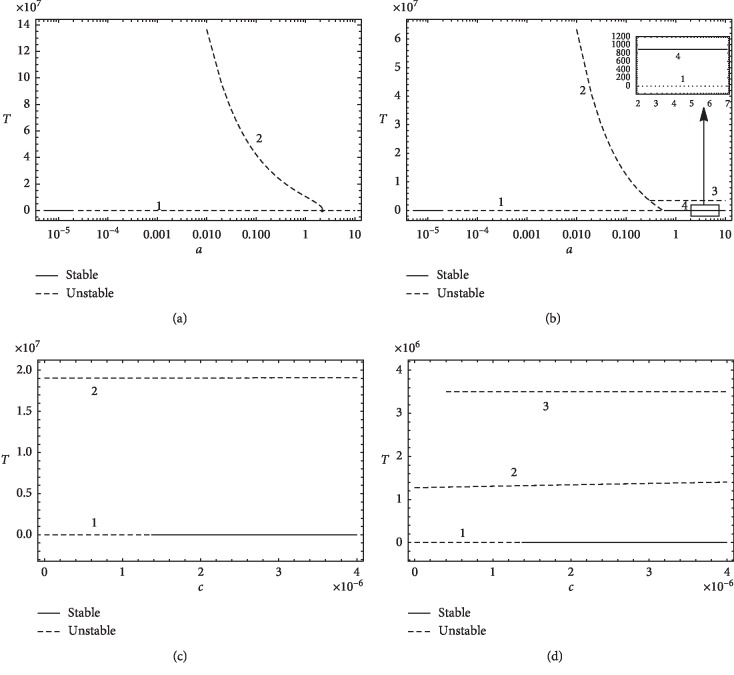
Bifurcation diagrams for the two patients: bifurcation diagram of (a) parameter *a* with first patient's data, (b) parameter *a* with second patient's data, (c) parameter *c* with first patient's data, and (d) parameter *c* with second patient's data.

First, the value of parameter *a* is changed from 0 to 10. Equilibrium points are monitored, and then bifurcation diagrams are plotted. In Figure 7(a), the bifurcation diagram for parameter *a* with first patient's data is illustrated. For *a* < 2.02257 × 10^−5^, there is only one equilibrium point at which *T*=0 and the system is stable. The value *a*=2.02257 × 10^−5^ is a bifurcation point because the system is turned into unstable after it. For 0.01 ≤ *a* ≤ 2.33, there is another equilibrium point due to the nonzero tumor case with zero cytokine. This point moves on curve 2 according to the value of *a*, and it is always unstable. The boundaries of curve 2, *a*=0.01 and *a*=2.33, are bifurcation points because this equilibrium point is created at *a*=0.01 and vanished at *a*=2.33. For *a* > 2.33, there is only one equilibrium point at the zero tumor state and it is unstable.

In Figure 7(b), the bifurcation diagram for the parameter *a* with second patient's data is plotted. It shows that, for *a* < 2.02257 × 10^−5^, there is only one equilibrium point at the zero tumor case and the system is stable. The value *a*=2.02257 × 10^−5^ is a bifurcation point because the system is turned into unstable after it. For 0.01 ≤ *a* ≤ 0.59, there is another equilibrium point due to the nonzero tumor case with zero cytokine. This point moves on curve 2 according to the value of *a* and is always unstable. The points *a*=0.01 and *a*=0.59 are bifurcation points. For the nonzero tumor with nonzero cytokine, there are two equilibrium points, illustrated by curves 3 and 4. The two equilibrium points created at the bifurcation points *a*=0.3 and *a*=0.6, respectively. Curve 3 is always unstable and exists for *a* ≥ 0.3. Curve 4 appears for *a* ≥ 0.6, and it represents the case with small size tumor and is always stable. A healthy state might be one which maintains the system at this stable low-tumor size level. Parameter *a* can be altered through treatments such as vaccination, and the tumor can be abolished/reduced by surgery or radiation and then immunotherapy and/or chemotherapy to reach the attraction zone of the tumor-free equilibrium state in the stable region of curve 1.

Bifurcation analysis is then performed for parameter *c*. It is changed from 0 to 4 × 10^−6^.  Figure 7(c) shows that, for first patient's data, there are two equilibrium points for all values of *c*. The equilibrium point moving on curve 2 for the nonzero tumor is always unstable. However, the equilibrium point moving on curve 1 for the zero tumor is unstable for *c* < 1.365 × 10^−6^ and stable for *c* > 1.365 × 10^−6^. The point *c*=1.365 × 10^−6^ is a bifurcation point.

Figure 7(d) shows that, for second patient's data, there are two equilibrium points for *c* < 0.4 × 10^−6^ and three equilibrium points for *c* ≥ 0.4 × 10^−6^. The equilibrium points moving on curves 2 and 3 are always unstable; however, the equilibrium point moving on curve 1 is unstable for *c* < 1.356 × 10^−6^ and stable for *c* > 1.356 × 10^−6^. The point *c*=1.356 × 10^−6^ is a bifurcation point. Point *c*=0.4 × 10^−6^ at which curve 3 is created is a bifurcation point also. It can be deduced that changing the value of parameter *c* leads to changing the stability of the zero tumor case only. For other equilibrium points, changing *c* is not practically effective because other points are unstable. So, surgery is slight effective.

Bifurcation analysis performed in this section shows that stability of the tumor can be controlled by changing patient's parameters. It is important to perform this analysis before deciding the treatment strategy and thus avoiding tumor recurrence.

## 4. Conclusions

In this study, a new mathematical model has been proposed in the form of a system of nonlinear ordinary differential equations to study the interaction between tumor cells and some immune cells. Effects of natural killer cells, circulating lymphocytes, CD8^+^T cells, and CD4^+^T cells on cancer cells have been investigated. Traditionally, CD4^+^T cells are considered helper cells that activate CD8^+^T cells. However, in the current study, the indirect role of CD4^+^T cells in killing tumor cells by secreting cytokines has been considered. Due to stiffness of the system of nonlinear ordinary differential equations, the model has been solved numerically using the method of Adams predictor-corrector with Adams–Bashforth predictor and Adams–Moulton corrector. The Adams method is usually used to solve a single nonlinear ordinary differential equation; however, in the current study, it is used to solve a system of nonlinear ordinary differential equations.

Three cases have been studied: first, the no-treatment case where interaction between immune cells and tumors with different sizes has been discussed. The results reveal that the ability of the patient's body to overcome tumor cells depending upon the patient's parameters, initial tumor size, and initial immune cells populations. For large tumors, the immune system alone fails to overcome the tumor and needs the intervention of treatment. Second, the case of continuous immunotherapy is considered where the role of CD4^+^T cells has been found to be very important and significant. In some cases, adoptive CD4^+^T cell therapy can be an alternative to combined CD8^+^T cells and cytokine therapy. In other cases, combined CD8^+^T cells and cytokine therapy is not enough to eliminate the tumor and introducing CD4^+^T cells is necessary. Third, the case of pulsed chemotherapy and immunotherapy is considered where CD4^+^T cell therapy has shown to be an alternative to chemotherapy in some cases. In other cases, specific pulses of chemotherapy alone could not be enough for treatment and immunotherapy could be introduced in order to completely eradicate the tumor. For large tumors, previous models abolished the cancer cells using intensive combined chemotherapy, CD8^+^T cells, and cytokine. In the current study, the same tumor has been abolished with a lower dose of chemotherapy combined with CD4^+^T cell therapy.

Stability analysis has been performed for the mathematical model. A condition for the stability of the zero tumor point has been deduced, and it depends on some immunological parameters of the patient. The stability analysis has answered the question of whether the tumor can recur after reaching size zero or not. If *a* − (*ceα*)/(*fβ*) < 0, then the zero tumor state will be a stable state and the tumor cannot recur. The results reveal that all equilibrium points are unstable for both the first and the second patients. This means that the size of the tumor is uncontrollable; i.e., it could increase without a limit. Also, after reaching the zero tumor equilibrium point using treatment, recurrence of the tumor can occur unless every single tumor cell is killed. We would like here to emphasize that the previously mentioned conclusions about the three studied cases would not be disturbed by the fact that the equilibrium points are unstable. For instance, it is still valid that CD4^+^T cells can replace chemotherapy or at least reduce its amount and, hence, spare patients the complications of chemotherapy-induced toxicities and side effects.

Finally, bifurcation analysis has been performed to show the effect of changing patient's parameters on the stability. Bifurcation analysis has been considered for parameters *a* and *c* with the data for the first patient and the second patient. It has specified the values of *a* and *c* for the system to be stable, and hence, tumor cannot recur after reaching the zero tumor case. The analysis has also shown that, for the second patient, there is a nonzero small amount of tumor case which is stable. Bifurcation analysis provides a powerful tool to control the stability of the tumor by changing patient's parameters.

The proposed model offers a powerful method for predicting the body response to immunotherapy and/or chemotherapy. Thus, the model provides a valuable tool for planning patients' treatment intervention strategies.

## Figures and Tables

**Table 1:** Description and values of patient's parameters.

Parameter (unit)	Description and sources	First patient	Second patient
*a* (day^−1^)	Tumor growth rate [40, 42].	4.31 × 10^−1^	4.31 × 10^−1^
*a*_1_ (cells)	Half saturation constant of the tumor killing rate [46].	1 × 10^5^	1 × 10^5^
*b* (cell^−1^)	1/b is the tumor carrying capacity [40, 42].	1.02 × 10^−9^	1.02 × 10^−9^
*c* (cell^−1^ · day^−1^)	Fractional no ligand-transduced tumor cell kill by natural killer cells [40, 42, 52].	6.41 × 10^−11^	6.41 × 10^−11^
*c*_1_ (cell · day^−1^)	Maximum tumor killing rate by cytokine [44, 46].	0.2	0.2
*d* (day^−1^)	Saturation level of fractional tumor cell kill by CD8^+^T cells. Primed with ligand transduced cells, challenged with ligand transduced [40, 42, 52].	2.34	1.88
*e* (day^−1^)	Fraction of circulating lymphocytes that become natural killer cells [40, 42].	2.08 × 10^−7^	2.08 × 10^−7^
*f* (day^−1^)	Death rate of natural killer cells [40, 42].	4.12 × 10^−2^	4.12 × 10^−2^
*g* (day^−1^)	Maximum natural killer cells recruitment by ligand transduced tumor cells [40, 42, 52].	1.25 × 10^−2^	1.25 × 10^−2^
*g*_*i*_ (cell^2^)	Steepness of CD8^+^T cell recruitment curve by cytokine [40, 42].	2 × 10^7^	2 × 10^7^
*h* (cell^2^)	Steepness coefficient of the natural killer cell recruitment curve [40, 42].	2.02 × 10^7^	2.02 × 10^7^
*j* (day^−1^)	Maximum CD8^+^T cell recruitment rate. Primed with ligand transduced cells, challenged with ligand transduced cells [40, 42, 52].	2.49 × 10^−2^	2.49 × 10^−2^
*k* (cell^2^)	Steepness coefficient of the CD8^+^T cell recruitment curve [40, 42, 52].	3.66 × 10^7^	5.66 × 10^7^
*K*_*T*_ (day^−1^)	Fractional tumor cells kill by chemotherapy [40, 42].	9 × 10^−1^	9 × 10^−1^
*K*_*N*_ (day^−1^)	Fractional natural killer cells kill by chemotherapy [40, 42].	6 × 10^−1^	6 × 10^−1^
*K*_*L*_ (day^−1^)	Fractional CD8^+^T cells kill by chemotherapy [40, 42].	6 × 10^−1^	6 × 10^−1^
*K*_*C*_ (day^−1^)	Fractional circulating lymphocytes cells kill by chemotherapy [40, 42].	6 × 10^−1^	6 × 10^−1^
*l* (none)	Exponent of fractional tumor cell kill by CD8^+^T cells. Primed with ligand transduced cells, challenged with ligand transduced cells [40, 42].	2.09	1.81
*m* (day^−1^)	Death rate of CD8^+^T cells [40, 42].	2.04 × 10^−1^	9.12
*p* (cell^−1^ · day^−1^)	Natural killer cell inactivation rate by tumor cells [40, 42].	3.42 × 10^−6^	3.59 × 10^−6^
*p*_*i*_ (day^−1^)	Maximum CD8^+^T cell recruitment rate by cytokine [40, 42].	1.25 × 10^−1^	1.25 × 10^−1^
*q* (cell^−1^ · day^−1^)	CD8^+^T cell inactivation rate by tumor cells [40, 42].	1.42 × 10^−6^	1.59 × 10^−6^
*r*_1_ (cell^−1^ · day^−1^)	Rate of which CD8^+^T cells are stimulated to be produced; as a result, tumor cells killed by natural killer cells [40, 42].	1.1 × 10^−7^	1.1 × 10^−7^
*r*_2_ (cell^−1^ · day^−1^)	Rate of which CD8^+^T cells are stimulated to be produced; as a result, tumor cells interaction with circulating lymphocytes [40, 42].	6.5 × 10^−11^	6.5 × 10^−11^
*s* (none)	Steepness coefficient of tumor-CD8^+^T cell lysis term D. Primed with ligand transduced cells, challenged with ligand transduced [40, 42].	8.39 × 10^−2^	5.12 × 10^−1^
*u* (cell^−2^ · day^−1^)	Regulatory function by natural killer cells of CD8^+^T cells [40, 42].	3 × 10^−10^	3 × 10^−10^
*α* (cell · day^−1^)	Constant source of circulating lymphocytes [40, 42].	7.5 × 10^8^	5 × 10^8^
*α*_1_ (cells)	Half saturation constant of the CD4^+^T cells production rate [46, 53].	1 × 10^3^	1 × 10^3^
*α*_2_ (cells)	Half saturation constant of cytokine production rate [44, 46].	1 × 10^3^	1 × 10^3^
*β* (day^−1^)	Natural death and differentiation of circulating lymphocytes [40, 42].	1.2 × 10^−2^	8 × 10^−3^
*β*_1_ (cell · day^−1^)	Maximum CD4^+^T cells production rate [44, 46].	0.835	0.835
*β*_2_ (cell^−1^ · day^−1^)	Maximum production rate of cytokine [44, 46].	5.4	5.4
*γ* (day^−1^)	Rate of chemotherapy drug decay [40, 42].	9 × 10^−1^	9 × 10^−1^
*μ*_1_ (day^−1^)	Natural death rate of CD4^+^T cells [45, 46].	1 × 10^−1^	1 × 10^−1^
*μ*_*i*_ (day^−1^)	Rate of cytokine decay [40, 42].	10	10
*δ*_2_ (cell^−1^ · day^−1^)	Loss rate of CD4^+^T cells due to interaction with tumor cells [44, 46].	1 × 10^−7^	1 × 10^−7^

**Table 2:** Stability results.

Patient	Equilibrium point number	( *T*_eq_ , *N*_eq_ , *L*_eq_ , *Y*_eq_ , *C*_eq_ , *I*_eq_ )	Stability
First patient	1	( 0, 315534 , 0 , 0 , 6.25 × 10^10^ , 0 )	Unstable
	2	(1.90609 × 10^7^, 199.335 , 2.82461 × 10^6^ , 0 , 6.25 × 10^10^ , 0 )	Unstable
Second patient	1	( 0, 315534 , 0 , 0 , 6.25 × 10^10^ , 0 )	Unstable
	2	(1.27423 × 10^6^, 2824.14 , 450073 , 0 , 6.25 × 10^10^ , 0 )	Unstable
	3	(3.50643 × 10^6^, 1030.37 , 952787, 4.55393 × 10^6^, 6.25 × 10^10^, 2.45842 × 10^6^ )	Unstable

## Data Availability

Previously reported human data were used to support this study and are available in Table 1. These prior studies are cited at relevant places within Table 1.
